# Methylmap: visualization of modified nucleotides for large cohort sizes

**DOI:** 10.1186/s12859-025-06106-3

**Published:** 2025-03-26

**Authors:** Elise Coopman, Svenn D’Hert, Rosa Rademakers, Wouter De Coster

**Affiliations:** 1 VIB Center for Molecular Neurology, VIB, Universiteitsplein 1, 2610 Wilrijk, Antwerp, Belgium; 2 Department of Biomedical Sciences, University of Antwerp, Antwerp, Belgium; 3 Department of Neuroscience, Mayo Clinic, Jacksonville, FL USA

**Keywords:** Modifications, Visualization, Methylation, Epigenetics, 1000 Genomes, ONT

## Abstract

**Background:**

Over the years, there has been growing interest in epigenetics, where nucleotide modifications are increasingly recognized for their roles in health and disease. Understanding methylation patterns at the nucleotide level has become pivotal for advancing this field. However, visualizing these modifications, particularly in cohorts of more than a few individuals, remains a challenge.

**Results:**

Here, we present methylmap, a tool developed to visualize modified nucleotide frequencies for regions of interest, specifically optimized for cohort sizes with more than a few individuals. Furthermore, methylmap features the visualization of the haplotype-specific methylation status of 226 individuals of the 1000 Genomes Project ONT Sequencing Consortium, sequenced using the Oxford Nanopore Technologies PromethION. This resource provides the research community with a comprehensive and complete overview of genome-wide methylation patterns.

**Conclusions:**

Methylmap offers an easy-to-use platform to facilitate epigenetic research. It is available both as a web application at https://methylmap.bioinf.be and as a command-line tool through Bioconda and PyPI. As such, we provide a valuable resource for advancing the understanding of epigenetic modifications in health and disease.

**Supplementary Information:**

The online version contains supplementary material available at 10.1186/s12859-025-06106-3.

## Background

In recent years, epigenetics has become a crucial topic of interest for understanding biological functions. Nucleotide modifications, particularly 5-methylcytosine (5mC) in eukaryotes, exhibit diverse physiological functions such as the regulation of gene expression, including genomic imprinting and X-chromosome inactivation, as well as repression of transposons [1]. These modifications are known to be altered in various disorders, including cancer, neurological diseases, and autoimmune diseases [2, 3].

While chemical and enzymatic methods, in combination with short read-sequencing, have been and continue to be widely applied to investigate nucleotide modifications, recent advancements in single-molecule long-read sequencing technologies such as Oxford Nanopore Technology (ONT) and Pacific Biosciences (PacBio) Single-Molecule Real-Time (SMRT) sequencing are starting to revolutionize the study of (epi)genetics [4]. These technologies support the simultaneous detection of multiple nucleotide modifications, such as methylation and hydroxymethylation. Furthermore, long-read sequencing enables the phasing of sequencing reads over long distances, enabling the ability to assign reads to specific haplotypes—the genetic variants inherited together on a single chromosome. This allows for the resolution of allele-specific modification information across large genomic regions, providing insights into how genetic and epigenetic variation influence each other [5, 6]. Long-read sequencing technologies are now increasingly applied in population-scale (epigenetic) sequencing projects, requiring the development of software tools to accommodate large cohort sizes [7]. Several tools for visualization of nucleotide modification patterns in one or a limited number of individuals are available [8–11]. However, to our knowledge, no software is suitable for visualizing nucleotide modifications in larger cohorts.

Over the years, the establishment of publicly available databases has revolutionized the research field, providing investigators with unprecedented access to extensive resources. With the arrival of the 1000 Genomes Project ONT Sequencing Consortium [12], a valuable resource of modification data became available for the research community. Easy and efficient access to this information is advantageous for accelerating research and improving the interpretation of epigenetic discoveries.

In this paper, we present methylmap, a tool with a dual-purpose application to enhance the field of epigenetic research. Methylmap allows users to visualize the haplotype-specific methylation status of individuals of the 1000 Genomes Project ONT Sequencing Consortium. Furthermore, methylmap enables users to visualize their own datasets, including those from large cohorts, offering powerful insights into nucleotide modification data.

### Implementation

We developed methylmap, a tool focused on visualizing nucleotide modification data. Methylmap is available as an easy-to-use web application and as a command line tool. Both the methylmap web application and the methylmap command line tool enable visualization of your own modification data for genomic regions of interest. The tool is specially tailored to efficiently handle datasets with significantly more individuals than existing visualization tools, which typically support visualization of only a limited number of individuals. Easy and efficient visualization of the haplotype-specific methylation of individuals of the 1000 Genomes Project ONT Sequencing Consortium is possible through the methylmap web application. To ensure homogeneity in the modification data, individuals were selected to have basecalls and modification detection (5-methylcytosine-guanine (5mCG) and 5-hydroxymethylcytosine-guanine modifications (5hmCG)) with Dorado (ONT). This led to the inclusion of 226 individuals, corresponding to 452 haplotypes. The BAM files were processed with the 1000 Genomes_snakemake.smk pipeline available in the methylmap GitHub repository. In short, this pipeline downloads the BAM files and extracts the per-base haplotype-specific methylation status with modkit (ONT). To generate a tab-separated output that focuses on cytosine bases followed by guanine bases and provides the information per haplotype with separate H1 and H2 files, the following parameters were used: –only-tabs, –partition-tag HP, –prefix H, and –cpg. Next, the files are split into smaller sections (size 25,000,000 basepairs) to manage memory efficiently and reorganized to create modification frequency tables with methylation frequencies per haplotype per genomic position. These modification frequency tables are merged together and sorted for genomic position, resulting in an overview modification frequency table with epigenome-wide (rows) methylation frequencies over 452 haplotypes (columns).

Methylmap is written in Python as a Dash application, providing a user-friendly web interface that enables real-time adjustment of input parameters. The methylmap web application allows for uploading modification data in a tab-separated table format (.tsv or.tsv.gz) with modification frequencies, where each column represents an individual or haplotype, and each row a genomic position (file size limit: 100 MB). Methylmap makes use of tabix [13] to facilitate the fast and efficient retrieval of data from a genomic region of interest. Starting from BAM or CRAM files with MM and ML tags, or tab-separated files from the nanopolish methylation caller [14], users can create a modification frequency table with the multiparsetable.py script provided on the methylmap GitHub page. This script supports fast processing of BAM/CRAM files using multithreading. The methylmap command line tool is sequencing technology-agnostic and directly supports input from BAM, CRAM, or nanopolish files or modification data in a tab-separated table.

For insights into gene or transcript structure for a genomic region of interest, methylmap offers the visualization of an annotation track supported by a GFF3 file. This feature can be accessed via the –gff argument in the command-line interface. Additionally, the methylmap web application provides built-in annotation files, including human annotation (GENCODE Release 46 for GRCh38.p14 comprehensive gene annotation in GFF3 format) and mouse annotation (GENCODE Release M36 for GRCm39 comprehensive gene annotation in GFF3 format) [15]. The set of provided annotation files in the methylmap web application will be expanded upon users' request.

Additionally, methylmap supports the option to perform hierarchical clustering on the modification frequencies, visualized by a dendrogram, using the Plotly figure factory ‘create_dendrogram’ module. When the hierarchical clustering option is selected, individuals or haplotypes with 40% or more missing data are first removed to prevent incomplete data from affecting the clustering process. Given that modifications are known to be coregulated, the interpolation function from the pandas package is then applied to estimate and fill in missing data. The linear interpolation method fills missing values using neighboring data from the same individual or haplotype, with greater weight given to closer positions for more accurate imputation. Finally, any individuals or haplotypes with missing values remaining after interpolation are subsequently removed, and the displayed heatmap will visualize the imputed data.

Methylmap depends on the pandas [16], numpy [17], plotly/dash [18] modules and modkit (ONT). The methylmap web application is available at https://methylmap.bioinf.be. The methylmap command-line tool is available through bioconda and PyPI.

## Results

Methylmap is a tool that enables epigenetic research to visualize modification data from various data types. It is especially suited for cohort sizes with a substantial number of individuals, as demonstrated by the ability to visualize the 452 haplotypes of 226 individuals of the 1000 Genomes ONT Sequencing Consortium [12]. Additionally, we successfully tested methylmap by visualizing an in-house dataset with methylation information across 698 haplotypes.

Methylmap is available as a command-line tool through bioconda and PyPI and has a web application available at methylmap.bioinf.be. The user-friendly web application requires no expertise in bioinformatics, ensuring its accessibility to researchers of all backgrounds.

**Fig. 1:**
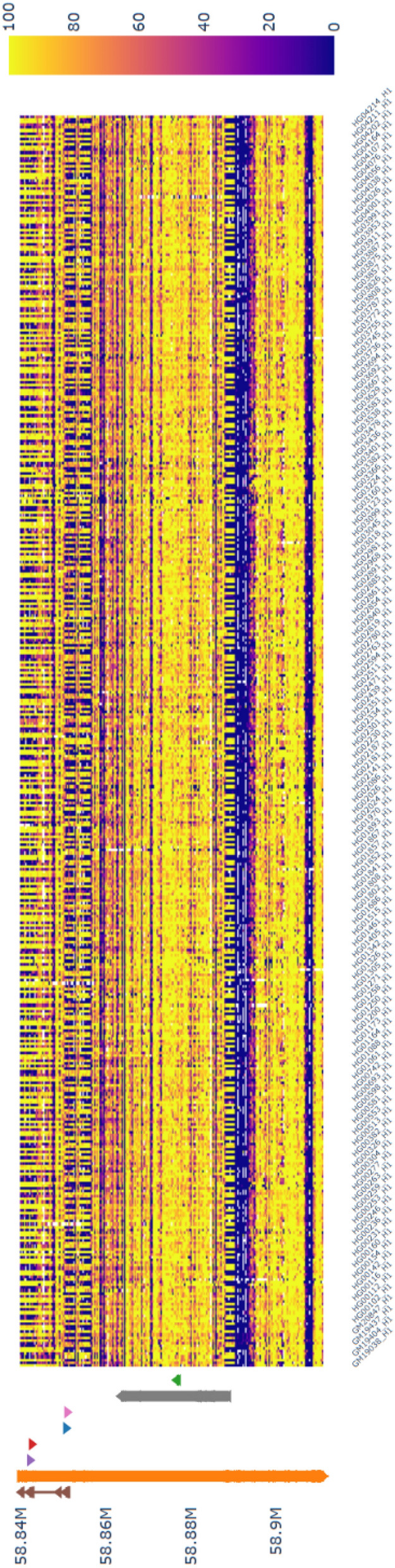
Methylation pattern of *GNAS* of individuals in the 1000 genomes project ONT sequencing consortium [12]. Methylation of the known imprinted gene *GNAS* of 452 haplotypes from 226 individuals of the 1000 genomes ONT sequencing consortium showing the alternating haplotype imprinting pattern. The heatmap shows high methylation frequencies in yellow and low methylation frequencies in purple. On the left side, the annotation of the region shows gene exon structure

Additionally, the methylmap web application serves as a resource for easy access to haplotype-specific methylation patterns across 226 individuals of the 1000 Genomes Project ONT Sequencing Consortium, offering a valuable resource for investigating epigenetic variation. For example, methylmap visualizes the haplotype-specific methylation pattern at the *GNAS* locus (chr20:58,839,718–58,911,192) from this dataset (Fig. 1), revealing known imprinted regions [19] that alternate by haplotype, as shown in the heatmap, with an annotation track highlighting the gene-exon structure. To demonstrate methylmap's technology-agnostic capability, we also visualize the same gene locus in the reduced representation bisulfite sequencing (RRBS) dataset from the Cancer Cell Line Encyclopedia in Supplementary Fig. 1.

Furthermore, researchers can use the implemented methylation data from the 1000 Genomes ONT Sequencing Consortium in methylmap to validate the significance of differentially methylated regions identified in their epigenetic studies. By comparing methylation patterns of a specific region of interest across diverse individuals, methylmap helps to distinguish true findings from those driven by high inter-individual variability. For instance, *GFPT2*, previously identified as a highly variable methylated region [20], shows inter-individual variability in the methylation data of the 1000 Genomes Project ONT Sequencing Consortium (Supplementary Fig. 2). Methylmap helps determine whether the observed variation is significant or due to intrinsic methylation variability, enhancing the robustness of methylation findings in epigenetic research.

The development of methylmap provides the research community with valuable resources for exploring epigenetic modifications and their role in various biological processes and diseases.

## Conclusions

In recent years, increased interest in epigenetic modifications has resulted in extensive developments in technologies that have made it possible to perform population-scale epigenetic studies. To support these efforts, we developed methylmap, a tool with two key functions: visualizing modification frequencies across large cohorts and providing an easy and efficient resource for consulting haplotype-specific methylation patterns of 226 individuals of the 1000 Genomes Project ONT Sequencing Consortium. In the future, this resource can be expanded to include additional datasets as they become available. Methylmap is technology-agnostic, requiring a tab-separated modification frequency input table as input via its web application. The methylmap command-line tool supports the direct input of BAM/CRAM files, nanopolish input files, or a tab-separated modification frequency table. With its current features and potential for future expansion and improvement, methylmap is designed to be a versatile tool for the epigenetics research community.

### Availability and requirements

- Project name: methylmap
- Project home page: https://methylmap.bioinf.be
- Operating systems(s): CLI: MacOS, Linux, Windows Subsystem for Linux (WSL); web-tool: platform-independent
- Programming language: Python
- Other requirements: Python 3 or higher, modkit (ONT)
- License: MIT
- Any restrictions to use by non-academics: None

## Supplementary Information

Supplementary Material 1.

## Data Availability

The original sequencing data of the 1000 Genomes Project ONT Sequencing Consortium is available at https://s3.amazonaws.com/1000g-ont/index.html?prefix=ALIGNMENT_AND_ASSEMBLY_DATA/FIRST_100/IN-HOUSE_MINIMAP2/HG38/ [12]. The original RRBS data from the Cancer Cell Line Encyclopedia is available at the depmap portal at Data | DepMap Portal [21, 22].

## Abbreviations

5mC: 5-Methylcytosine
5mCG: 5-Methylcytosine-guanine
5hmCG: 5-Hydroxymethylcytosine-guanine
ONT: Oxford nanopore technologies
RRBS: Reduced representation bisulfite sequencing
