# Supplementary material for: Methylmap: visualization of modified nucleotides for large cohort sizes
Source: BMC Bioinformatics. 2025 Mar 26;26:91. doi: 10.1186/s12859-025-06106-3 (PMC11948879; doi:10.1186/s12859-025-06106-3)

**Supplementary Figure 1. Methylation pattern of *GNAS* in 843 cell lines part of the Cancer Cell Line Encyclopedia.** Methylation of *GNAS* in 843 cell lines from the Cancer Cell Line Encyclopedia, based on RRBS data. The heatmap shows high methylation frequencies in yellow and low methylation frequencies in purple.


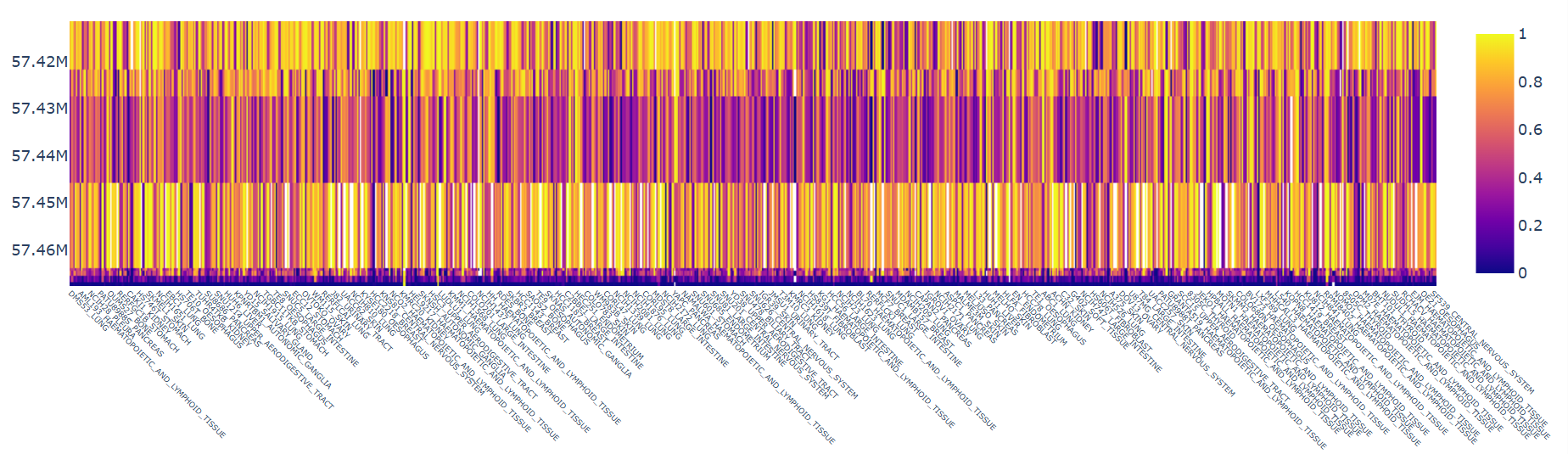


**Supplementary Figure 2. Methylation pattern of *GFPT2* of individuals in the 1000 Genomes Project ONT Sequencing Consortium.** Methylation in the *GFPT2* region of 452 haplotypes from 226 individuals of the 1000 Genomes ONT Sequencing Consortium shows high variability in methylation patterns between individuals. The heatmap shows high methylation frequencies in yellow and low methylation frequencies in purple. On the left side, the annotation of the region shows gene exon structure.


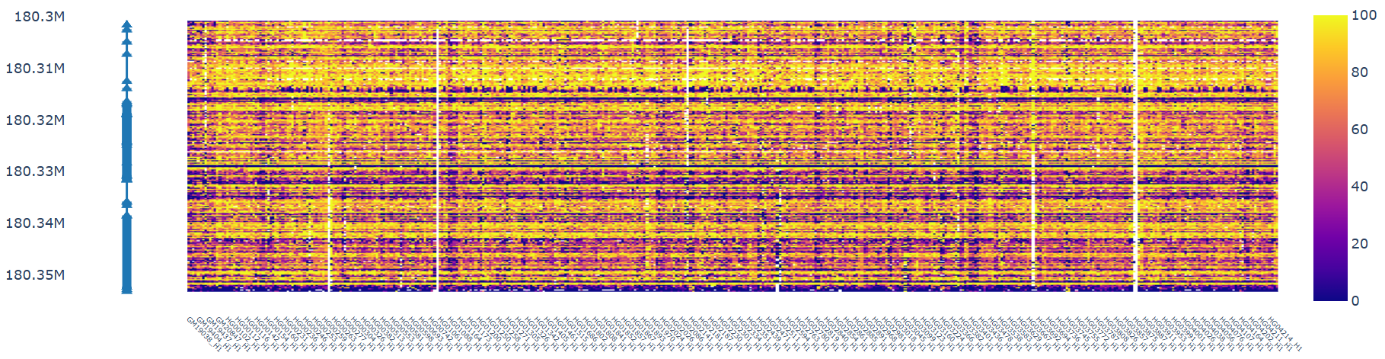

Supplement: Supplementary file 1 — Supplementary Material 1. [file 12859_2025_6106_MOESM1_ESM.docx]
